# The impact of *Aloe vera* and licorice extracts on selected mechanisms of humoral and cell-mediated immunity in pigeons experimentally infected with PPMV-1

**DOI:** 10.1186/s12917-018-1467-3

**Published:** 2018-05-02

**Authors:** Daria Dziewulska, Tomasz Stenzel, Marcin Śmiałek, Bartłomiej Tykałowski, Andrzej Koncicki

**Affiliations:** Department of Poultry Diseases, Faculty of Veterinary Medicine, University of Warmia and Mazury in Olsztyn, ul. Oczapowskiego 13/14, 10-719 Olsztyn, Poland

**Keywords:** *Aloe vera*, Flow cytometry, Gene expression, Herbal extracts, Licorice, Pigeons, PPMV-1

## Abstract

**Background:**

The aim of the study was to evaluate the impact of herbal extracts on selected immunity mechanisms in clinically healthy pigeons and pigeons inoculated with the pigeon paramyxovirus type 1 (PPMV-1). For the first 7 days post-inoculation (dpi), an aqueous solution of *Aloe vera* or licorice extract was administered daily at 300 or 500 mg/kg body weight (BW). The birds were euthanized at 4, 7 and 14 dpi, and spleen samples were collected during necropsy. Mononuclear cells were isolated from spleen samples and divided into two parts: one part was used to determine the percentage of IgM^+^ B cells in a flow cytometric analysis, and the other was used to evaluate the expression of genes encoding IFN-γ and surface receptors on CD3^+^, CD4^+^ and CD8^+^ T cells.

**Results:**

The expression of the IFN-γ gene increased in all birds inoculated with PPMV-1 and receiving both herbal extracts. The expression of the CD3 gene was lowest at 14 dpi in healthy birds and at 7 dpi in inoculated pigeons. The expression of the CD4 gene was higher in uninoculated pigeons receiving both herbal extracts than in the control group throughout nearly the entire experiment with a peak at 7 dpi. A reverse trend was observed in pigeons inoculated with PPMV-1 and receiving both herbal extracts. In uninoculated birds, increased expression of the CD8 gene was noted in the pigeons receiving a lower dose of the *Aloe vera* extract and both doses of licorice extracts. No significant differences in the expression of this gene were found between inoculated pigeons receiving both herbal extracts. The percentage of IgM^+^ B cells did not differ between any of the evaluated groups.

**Conclusions:**

This results indicate that *Aloe vera* and licorice extracts have immunomodulatory properties and can be used successfully to prevent viral diseases, enhance immunity and as supplementary treatment for viral diseases in pigeons.

## Background

Immunomodulation is the stimulation or suppression of immune responses in living organisms. Numerous substances, both natural and synthetic, exert effects on immunity. Natural immunomodulators include herbal preparations whose popularity continues to increase due to the decreasing effectiveness of antibiotics and other synthetic drugs [27].

The therapeutic properties of *Aloe vera*, also known as Barbados aloe (*Aloe barbadensis Miller*), have been recognized already in ancient times. *Aloe vera* is a succulent plant of the lily family (*Liliaceae*) [6]. The part of *Aloe vera* plants that plays the most important role in natural medicine are its leaves which are a rich source of latex and gel containing 98.5% to 99.5% water and 75 biologically active compounds [9]. *Aloe vera* gel also contains polysaccharides, including acemannan which is one of the most potent plant-derived immunomodulators. Acemannan binds to macrophage receptors and stimulates the synthesis of cytokines (interleukin 1 (IL-1), interleukin 6 (IL-6)) and tumor necrosis factor-alpha (TNF-α) [8, 11]. *Aloe vera* extract could also stimulate cell-mediated immunity (CMI). Vahedi et al. (2011) reported a higher percentage of CD4^+^ and CD8^+^ T cells in the peripheral blood of rabbits receiving *Aloe vera* extract [43]. *Aloe vera* extracts were also found to stimulate humoral immunity in chickens experimentally infected with the Newcastle disease virus (NDV) [26]. The discussed plant delivers numerous health benefits and exerts anti-inflammatory, antibacterial, antifungal and anti-carcinogenic effects due to the presence of anthraquinones, saccharides and antioxidant vitamins (A, C and E) [38].

Licorice (*Glycyrrhiza glabra*) is also a popular medicinal plant of the legume family (*Fabaceae*). It is valued mostly for its roots which contain 1% to 9% glycyrrhizic acid (glycyrrhizin) [15]. Glycyrrhizin is a potent immunomodulator which stimulates the production of interferon [1, 42] and the proliferation of regulatory (Treg) cells in mice [16]. Licorice extracts have been found to increase the phagocytic capacity of chicken granulocytes and mononuclear cells [12]. Similarly to *Aloe vera*, licorice exerts various types of antiviral activity. Licorice inhibits viral replication not only by becoming attached to the cell membrane and compromising the cells’ ability to undergo endocytosis, which prevents the virus from penetrating cells [46], but also by activating the NF-κB protein complex which plays a key role in regulating the immune response to infections and stimulates IL-8 secretion [34].

Medicinal herbs are widely used as functional additives in animal diets to improve the palatability and digestibility of feed [4, 21]. Herbal functional additives have various properties and can be used in the prevention and supplementary treatment of infectious diseases with different etiology. These properties can be directly attributed to herbal extracts’ ability to stimulate immune responses, which was observed in chickens [9]. For example, Liu et al. (2010) demonstrated that the addition of four herbal extracts, *Astragalus membranaceus, Codonopsis pilosula, Epimedium spp.* and *Glycyrrhiza uralensis,* to drinking water can enhance the immune response in immunosuppressed chickens with the reticuloendotheliosis virus [23]. Further evidence of the immunomodulatory effects of herbal extracts was provided by Latheef et al. (2017) who reported that *Withania somnifera*, *Tinospora cordifolia* and *Azadirachta indica* were capable of inhibiting the replication of the chicken infectious anemia virus and increasing the cell-mediated response of chickens against this virus [22]. However, the immunomodulatory effects of herbal extracts have never been investigated in domestic pigeons.

Viral diseases, in particular infections with the pigeon circovirus (PiCV) which exerts immunosuppressive effects, pose a serious problem in pigeon breeding [36]. Since a laboratory protocol for culturing PiCV under laboratory conditions has not been developed to date [10], the pigeon paramyxovirus type 1 (PPMV-1), the pigeon variant of NDV, is successfully used for experimental inoculation of pigeons [13, 28, 37, 39].

The course of an NDV infection can differ substantially, depending on the strain’s virulence [28]. Strain virulence also determines birds’ immune responses to infection or inoculation with live vaccines. The early immune response to a viral infection is influenced by innate immunity, a universal mechanism that protects living organisms against infections. Innate immunity relies on pattern recognition receptors (PRRs) which identify pathogen-associated molecular patterns (PAMPs). PRRs enable an organism to discriminate between non-self and self antigens. Toll-like receptors (TLRs) are a group of PRRs which play a key role in the initiation of immune responses [40]. TLRs are found on the surface of selected immune system cells, such as lymphocytes, heterophils and macrophages, and their stimulation constitutes a signal that activates non-specific and specific immune responses. In an in vitro study, the inoculation of chicken peripheral blood heterophils and mononuclear cells with NDV stimulated the production of interferon and nitric oxide (NO) [2]. Research has also demonstrated that the expression of genes encoding interferon α (IFN-α), IFN-ß, IL-1ß and IL-6 increased in chicken splenocytes inoculated with NDV [32]. However, the observed increase in expression was determined by the NDV strain and its virulence, and it was not induced by mild viral strains [20, 24].

Cell-mediated immunity associated with T cells, including cytokine-producing CD4^+^ lymphocytes and cytotoxic CD8^+^ lymphocytes (CTL), also plays an important role in the immune response to NDV. In chickens, a CMI response to NDV was observed already 2–3 days after inoculation with the vaccine virus strain [31]. Similarly to the innate immune response, the adaptive immune response is influenced by several factors, including strain virulence [30] and the breed of chickens exposed to vaccine and field isolates [7]. The humoral immune response, which involves the proliferation of B cells and the production of immunoglobulins M, Y and A (IgM, IgY, IgA), is also an important element of immunity against NDV [19]. Anti-NDV antibodies are detected in mucosal membranes of the upper respiratory tract and in blood already 6 days after infection or inoculation with an attenuated vaccine, and their concentrations peak 21–28 days after infection. The antibodies’ role is to neutralize the virus by binding to it and preventing it from adhering to host cells [3].

The influence of paramyxovirus infections on the immune response in birds has been studied extensively [19, 20, 24, 32]. However, very little is known about the influence of immunomodulatory herbal extracts on viral infections and immune responses in infected birds. A few studies have been conducted to investigate the immunomodulatory effects of *Aloe vera* and licorice extracts on birds infected with the avian paramyxovirus serotype-1 (APMV-1), and their results are limited to analyses of antibodies against this virus in chickens [26].

In view of alternative immunomodulation-based strategies and the scarcity of published information relating to the applicability of immunomodulatory herbal extracts in pigeons, the main aim of this basic research was to determine the influence of *Aloe vera* and licorice extracts on selected mechanisms of cell-mediated and humoral immunity in virus-inoculated pigeons. PPMV-1 was used as an experimental model because it is easy to culture under laboratory conditions. However, it should be noted that Newcastle disease is a notifiable disease that has to be legally reported to the authorities, and treatment of PPMV-1 infections in pigeons is not allowed.

## Methods

### Virus

Pigeons were infected with the pigeon paramyxovirus serotype-1 (PPMV-1/pigeon/Poland/AR3/95) obtained from the National Veterinary Research Institute in Puławy. The pathogenicity of the applied isolate was classified based on biological (calculation of the Intracerebral Pathogenicity Index (ICPI) for one-day-old SPF chickens) and molecular analyses (analysis of the amino acid sequence at the cleavage site in the fusion protein). The ICPI was 1.4, and the amino acid sequence at the cleavage site in the fusion protein was ^112^R-R-Q-K-R-F^117^. Based on those results, the virus was classified as a mesogenic pathotype.

### Plant extracts

#### Aloe vera

The *Aloe vera* extract was obtained by freeze/spray drying of aloe leaf juice. Five grams of the extract with maximum moisture content of 8% and bulk density of 0.3–0.6 g/1 ml were obtained from 1000 g of fresh *Aloe vera* juice.

#### Licorice

Dry licorice extract was obtained by spray drying an aqueous solution of licorice root, a registered feed additive (European Union Register of Feed Additives, group 2b: natural products – botanically defined: CAS 68916–91-6 FEMA 2629, CoE 218, pursuant to Regulation (EC) No 1831/2003). The extract contained 20% glycyrrhizic acid, and it was characterized by maximum moisture content of 3.6% and bulk density of 0.5 g/1 ml.

*Aloe vera* and licorice extracts were free of pathogenic bacteria such as *Escherichia coli, Staphylococcus aureus* and *Pseudomonas aeruginosa* in 10 g of the product. The contamination of the extract with selected pathogenic bacteria (*Escherichia coli, Staphylococcus aureus* and *Pseudomonas aeruginosa*) was determined in accordance with PN-EN ISO 6887–1 [29]. First, a 10% solution of the extract was prepared in the amount of 100 mL (conc. 10^− 1^), and it was used as the initial suspension that was diluted ten-fold to obtain a concentration of 10^− 5^. Using a sterile pipette, 1 mL of the sample from each dilution was transferred to the following culture media: MacConkey Agar No. 3, Columbia Agar with sheep blood plus and Mannitol salt agar. All culture media were obtained from the same manufacturer (Oxoid, UK), and all analyses were conducted in duplicate. The suspension was spread evenly with a sterile cell spreader, and the plates were incubated at a temperature of 37 °C for 24 h. Beginning with the first dilution, an increase in the CFU of the tested pathogenic bacteria was not observed on any of the plates after incubation.

#### Pigeons

One hundred twenty 8-week-old fantail pigeons were obtained from a private breeder. The flock in the breeding facility had not been vaccinated against PPMV-1 since 2008, and it was free of the infection. Before the experiment, cloacal swabs and blood samples were collected from all birds to rule out PPMV-1 infection with the use of the real-time PCR method described by Wise et al. (2004) and modified by Cattoli et al. (2009) and to determine the presence of antibodies against PPMV-1 with the use of the commercial ELISA test kit (IDEXX, USA) according to the method described by Stenzel et al. (2011) [5, 35, 45]. The birds were housed in isolated units in a PCL3 biosafety facility of the Department of Poultry Diseases, Faculty of Veterinary Medicine of the University of Warmia and Mazury in Olsztyn. The biosafety facility is equipped with a HEPA filtering system and an automated system for pressure control in corridors, bird units and hygiene stations to prevent contamination of experimental premises. Every group of pigeons was housed in a separate unit. The birds were administered seed mixtures and water ad libitum throughout the experiment.

### Experimental design

Pigeons were divided into 10 groups of 12 birds each. Pigeons from groups A1, B1, C1, D1 and K1 were inoculated oculonasally with 10^6^ EID_50_ of PPMV-1 at 100 μL per bird (applied to the nostril and the eye at 50 μL each). For the first 7 days post-inoculation (dpi), an aqueous solution of *Aloe vera* extract was administered daily per os at 300 mg/kg body weight (BW) (groups A and A1) or 500 mg/kg BW (groups B and B1), and an aqueous solution of licorice extract was administered at 300 mg/kg BW (group C, C1) or 500 mg/kg BW (group D, D1). Control group (K and K1) birds were orally administered 0.9% NaCl. At 4, 7 and 14 dpi, the birds were euthanized by intravenous administration of pentobarbital sodium at 70 mg/1 kg BW (Morbital, Biowet Puławy, Poland) after premedication by intramuscular injection of butorphanol tartrate at 4 mg/1 kg BW (Torbugesic, Zoetis, USA), and spleen samples were collected during an anatomopathological examination (Table 1). Mononuclear cells were isolated from spleen samples and divided into two parts: one part was used to determine the percentage of IgM^+^ B cells in a flow cytometric analysis, and the other was used in RNA extraction to evaluate the expression of genes encoding IFN-γ and surface receptors on CD3^+^, CD4^+^ and CD8^+^ T cells.

**Table 1:** Experimental design

Group	Day of experiment					
	1–7	8	9–15	12	15	22
		Experimental inoculation with PPMV-1, 10^6^ EID_50_	Once daily administration of:			
A	Adaptation to new conditions	–	*Aloe vera*, 300 mg/kg BW	Collection of spleen samples for molecular biology and flow cytometry analyses		
A1		+				
B		–	*Aloe vera*, 500 mg/kg BW			
B1		+				
C		–	licorice, 300 mg/kg BW			
C1		+				
D		–	licorice, 500 mg/kg BW			
D1		+				
K		–	0.9% NaCL			
K1		+				

### Isolation of mononuclear cells

Mononuclear cells were isolated from whole spleens using the manual Dounce tissue grinder (Kimble, USA) in 9 ml of a complete growth medium (RPMI – 1640, 10% fetal bovine serum (FBS), 1% MEM non-essential amino acids solution, 1% penicillin – streptomycin, 1% HEPES, 1% sodium pyruvate) (Sigma Aldrich, USA) and were filtered (70 μm mesh). A homogenous suspension was obtained, and centrifuged cell pellets (450 g for 10 min at 25 °C) were resuspended in 2.3 mL of a complete growth medium and gently layered on 2.5 mL of Histopaque-1077 (Sigma Aldrich, USA). After centrifugation (30 min, 400 g, at room temperature), the upper layer of the opaque interface containing mononuclear cells was carefully aspirated. Finally, the obtained mononuclear cells were washed twice and resuspended in 1 mL of PBS (phosphate-buffered saline) (Sigma Aldrich, USA). Cell concentrations and the percentage of viable cells were determined in the Vi-cell XR analyzer (Beckman Coulter, USA).

### Flow cytometry

Before the experiment, the cross-reactivity of Goat anti-Chicken IgM-FITC polyclonal antibodies (AbD Serotec, UK) was checked in pigeon lymphocytes. For this purpose, mononuclear cells were isolated from the thymus, bursa of Fabricius and peripheral blood. The antibodies’ cross-reactivity was characterized by 3.99%, 44.46% and 9.5% of IgM^+^ B cells isolated from the thymus, bursa of Fabricius and peripheral blood, respectively. The quantity of the tested antibodies (3 μg antibodies per one million cells) was determined experimentally in serial dilutions (1 to 5 μg antibodies per one million cells).

Thereafter, half a million mononuclear cells isolated from spleen samples were stained with 1.5 μg of the IgM^+^ polyclonal antibody for B cells. The samples were incubated in darkness on ice for 30 min. Next, the cells were twice rinsed in PBS, centrifuged at 400 g for 10 min, and the resulting pellets were suspended in 400 μL of PBS and analyzed with the use of the FACS Canto II (BD, USA) flow cytometer. Data were acquired in FACS Diva Software 6.1.3. (BD, USA). Cells were analyzed and immunophenotyped in FloJo 7.5.5 (Tree Star, USA).

### Real-time PCR

The number of mononuclear cells isolated from spleen samples was standardized to 5 × 10^6^ and used for RNA isolation with the use of the RNeasy Mini Kit (Qiagen, Germany) according to the manufacturer’s protocol. Genomic DNA remaining in the samples after RNA isolation was digested with deoxyribonuclease I (Sigma Aldrich, USA). RNA quality was evaluated in the 2100 Bioanalyzer (Agilent, USA). The concentrations of eluted RNA were measured with the NanoDrop 2000 spectrophotometer (Thermo Fisher Scientific, USA), and the samples were stored at − 80 °C until further analysis.

Reverse transcription was carried out with the High-Capacity cDNA Reverse Transcription Kit (Life Technologies, USA) according to the manufacturer’s recommendations. The concentration of RNA for the synthesis of complementary DNA (cDNA) was standardized to 0.5 μg per sample. The expression of the gene encoding IFN-γ and the genes encoding receptors on the surface of T cells (CD3, CD4 and CD8) was determined by real-time PCR. The reaction mixture for all analyzed genes had the following composition: 10 μL of the Power SYBR® Green PCR Master Mix (Life Technologies, USA), 1.8 μL of each 10 μM primer, 4.4 μL of RNase-free water, and 2 μL of cDNA. The primer sequences and the accession numbers of gene sequences used for designing the primers are presented in Table 2. The reaction was carried out under the following conditions: polymerase activation at 95 °C for 10 min, followed by 40 two-stage cycles: denaturation at 95 °C for 30 min, primer annealing and chain elongation at 60 °C for 60 s. The relative expression of each gene was calculated using the 2^−ΔΔCt^ method [25] normalized to efficiency corrections, expression levels of reference gene coding glyceraldehyde 3-phosphate dehydrogenase (GAPDH) and reference groups (K and K1) in GenEx 6.1.0.757 data analysis software (MultiD, Sweden).

**Table 2:** Primers used for real time PCR

Primer	Sequence 5′ - > 3′	Fragment size (bp)	Accession number
CD3 F	GCAATTTACGATGATCCCAGAG	112	XM_005500716.2
CD3 R	GCGTCCACTTCAATGCAATTC		
CD4 F	GAACGTGTGAATGGGACTCAGA	116	MG214789
CD4 R	GTCATTGTCTTCTATGAGGTGACA		
CD8 F	TTCATCTGGGTTCCCTTGGCA	97	MG214790
CD8 R	CTGCATCTTCGGCTCCTGGT		
IFNγ F	CTGACAAGTCAAAGCCGCAC	125	DQ479967.1
IFNγ R	AGTCATTCATCT GAAGCTTGGC		
GAPDH F	CCCTGAGCTCAATGGGAAGC	137	NM_001282835.1
GAPDH R	TCAGCAGCAGCCTTCACTAC		

### Statistical analysis

The significance of differences between the relative expression of IFN-ɣ, CD3, CD4 and CD8 genes and the percentage of IgM^+^ B cells were analyzed using the Kruskal-Wallis non-parametric test for independent samples. The analyzed factors were the experimental group and the day of the experiment. Differences were considered significant at a confidence level of 95% (*P* < 0.05).

## Results

### Expression of the IFN-ɣ gene

**Fig. 1:**
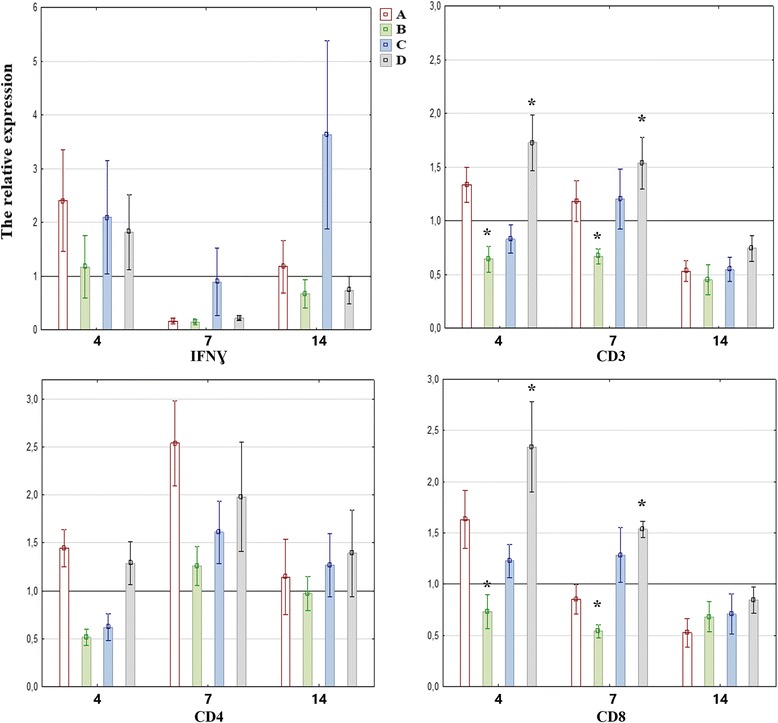
Mean relative expression of the genes encoding IFN-ɣ and CD3, CD4, CD8 receptors, in splenic mononuclear cells of pigeons administered herbal extracts, at 4, 7 and 14 dpi. The mean relative expression values above 1 (black line) in groups A-D indicate higher gene expression in comparison with the control group (K). The statistical differences between groups are marked with an asterisk. Error bars represent the standard error of the mean

**Fig. 2:**
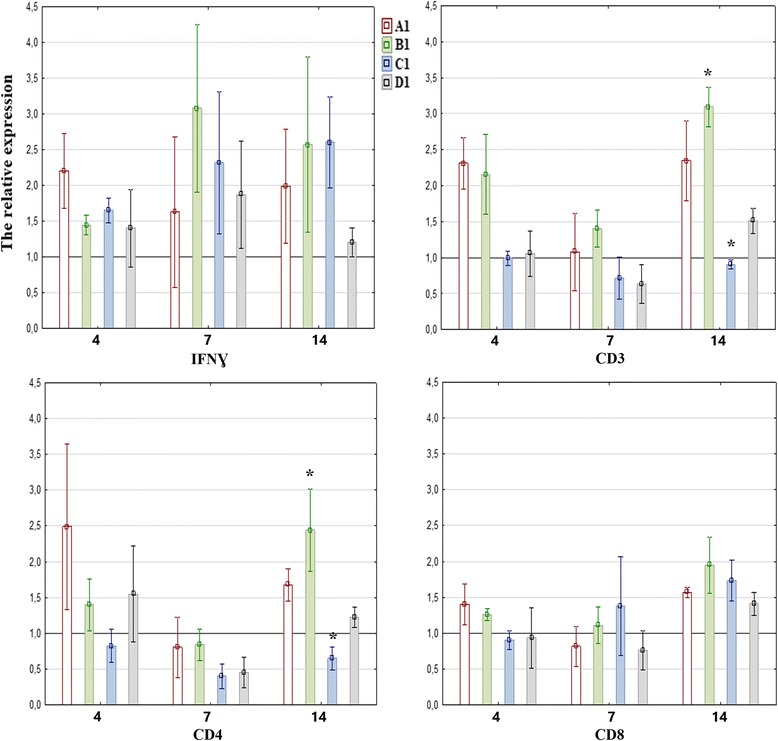
Mean relative expression of the genes encoding IFN-ɣ and CD3, CD4, CD8 receptors, in splenic mononuclear cells of pigeons inoculated with PPMV-1 and administered herbal extracts, at 4, 7 and 14 dpi. The mean relative expression values above 1 (black line) in groups A1-D1 indicate higher gene expression in comparison with the control group (K1). The statistical differences between groups are marked with an asterisk. Error bars represent the standard error of the mean

No significant differences in the expression of the gene encoding IFN-ɣ in mononuclear cells isolated from spleen samples were observed between the experimental groups during the experiment. The expression of the above gene was higher in groups A and C at 4 and 14 dpi, and in groups B and D at 4 dpi than in control group K (expression level > 1). In birds uninfected with PPMV-1, the lowest levels of IFN-ɣ gene expression were noted at 7 dpi (Fig. 1). In all inoculated birds receiving herbal extracts, the expression of the IFN-ɣ gene was higher than in control group K1 at 4, 7 and 14 dpi (Fig. 2).

### Expression of the CD3 gene

The expression of the gene encoding surface receptor CD3 in the mononuclear cells of group A and D birds at 4 and 7 dpi and group C birds at 7 dpi was higher than in the control group. In group B, the expression of the analyzed gene did not increase relative to group K throughout the experiment, and it was significantly lower than in group D at 4 and 7 dpi (*P* = 0.023 and *P* = 0.045, respectively) (Fig. 1). The expression of the CD3 gene was higher in groups A1 and B1 throughout the entire experiment, and in group D1 at 4 and 14 dpi than in the control group (K1). Group C1 pigeons were characterized by lower expression of the CD3 gene than group K1 birds throughout the experiment, and significantly lower expression of the CD3 gene than group B1 pigeons at 14 dpi (*P* = 0.006) (Fig. 2).

### Expression of the CD4 gene

No significant differences in the expression of the gene encoding surface receptor CD4 in mononuclear cells isolated from spleen samples were observed between pigeons from groups A-D. However, the expression of the above gene in groups A-D was higher than in the control group throughout the experiment. The only exceptions were pigeons from group B at 4 and 14 dpi and pigeons from group C at 4 dpi. The highest number of copies of the CD4 gene in groups A-D were detected at 7 dpi (Fig. 1). A reverse trend was noted in groups A1-D1 where CD4 gene expression was lowest at 7 dpi. At 14 dpi, the expression of the CD4 gene was significantly higher in group B1 than in group C1 (*P* = 0.011) (Fig. 2).

### Expression of the CD8 gene

In comparison with group K pigeons, the expression of the CD8 gene was higher only in groups A, C and D at 4 dpi, and in groups C and D at 7 dpi. The expression of the CD8 gene was significantly higher in group D at 4 and 7 dpi than in group B (P = 0.011 and *P* = 0.045, respectively) (Fig. 1). No significant differences in the number of copies of the CD8 gene were found between infected birds receiving herbal extracts and control group birds, but CD8 gene expression in the above experimental groups peaked at 14 dpi (Fig. 2).

### Flow cytometric analysis

**Fig. 3:**
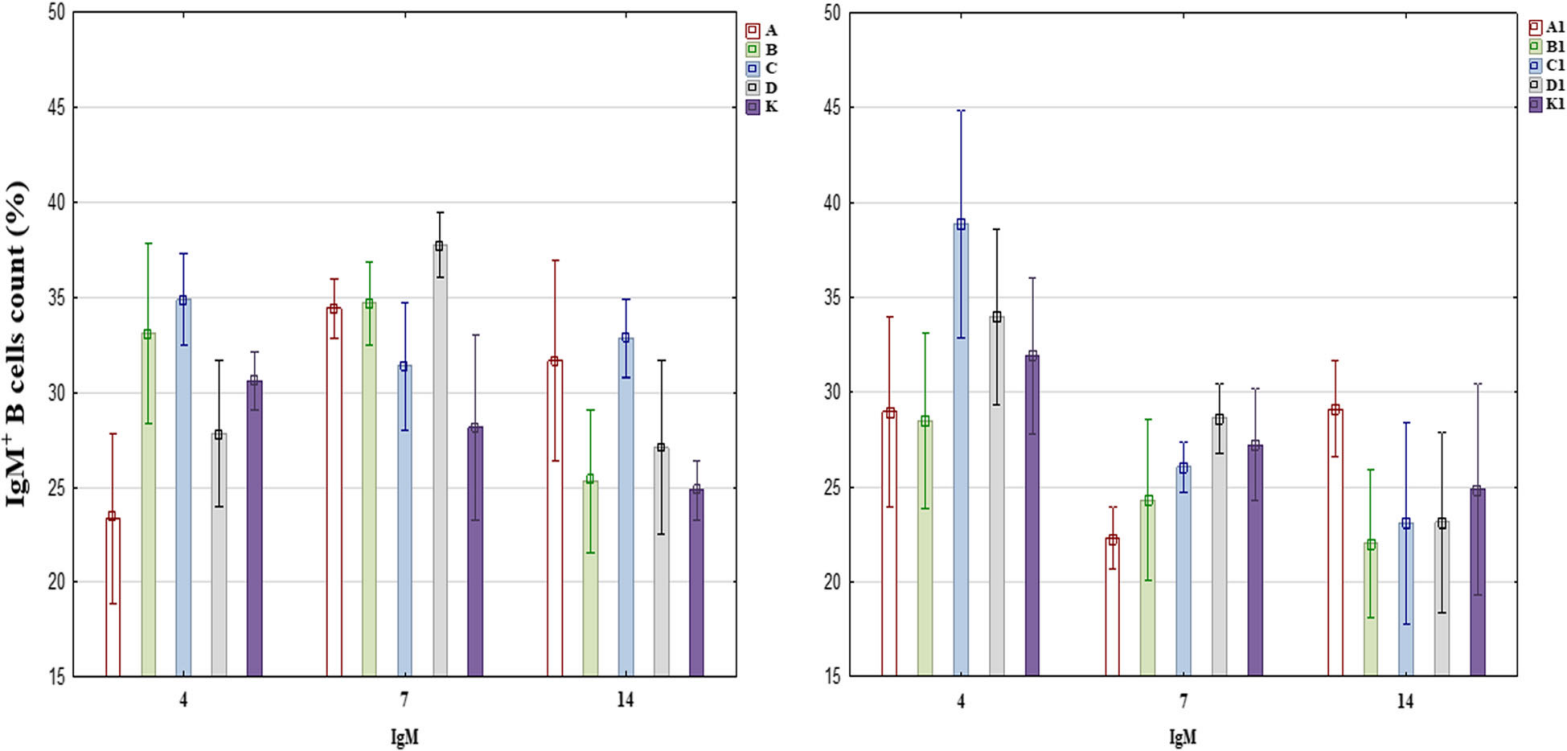
Percentage of IgM^+^ B cells at 4, 7 and 14 dpi in healthy pigeons administered herbal extracts (left) and in pigeons administered herbal extracts and inoculated with PPMV-1 (right)

Flow cytometry data are presented in Fig. 3. No significant differences in the percentage of IgM^+^ B cells were found between experimental groups or between sampling dates. The highest percentage of IgM^+^ B cells was noted at 7 dpi in groups A, B and D (34.4%, 34.67% and 37.72%, respectively). In comparison, in control group birds at 7 dpi, the percentage of IgM^+^ B cells was determined at 28.14% (group K) and 27.23% (group K1). The percentage of IgM^+^ B cells was lowest in groups A1, B1 and C1 at 7 dpi (22.30%, 24.33% and 26.05%, respectively) and in groups B1, C1 and D1 at 14 dpi (22.04%, 23.10% and 23.14%, respectively).

## Discussion

Immunomodulators are biological and synthetic substances which stimulate or suppress humoral and cell-mediated immune responses [18]. Antibiotics and synthetic drugs are increasingly replaced by plant-based immunomodulators in the treatment and prevention of animal diseases. Natural materials such as herbs, spices, essential oils and oleoresins are classified as phytogenic feed additives or phytobiotics [4, 44]. Phytobiotics are a rich source of biologically active compounds with a wide range of anti-carcinogenic, anti-inflammatory, antibacterial, antifungal and antiviral properties [17].

Diseases with a viral etiology pose a growing problem in pigeon breeding. This can be attributed mainly to pigeon rearing systems where infectious diseases spread rapidly due to the absence of biosecurity procedures. In view of the above, the present experiment was carried out to demonstrate the immunomodulatory properties of *Aloe vera* and licorice in the supplementary treatment of viral infections using PPMV-1 as an experimental model. Molecular and flow cytometry analyses were performed on mononuclear cells isolated from the spleen which is the first lymphoid organ to be colonized by pathogenic paramyxovirus strains during infection. [32]. The percentages of T cell subpopulations were not determined by flow cytometry due to the absence of monoclonal antibodies reacting with pigeon lymphocytes. The investigation conducted previously by Stenzel et al. (2011) with the use of flow cytometry was burdened with high methodological error because commercially available antibodies against chicken lymphocytes are characterized by minimal cross-reactivity with pigeon cells [35]. For this reason, a method for evaluating the expression of genes encoding surface receptors on CD3, CD4 and CD8 T cells was developed in this study as a reliable alternative to flow cytometry.

The results of the conducted research revealed that both herbal extracts had immunomodulatory properties which differed radically depending on the dose and the presence or absence of inoculation with PPMV-1. In uninfected birds, the analyzed herbal extracts stimulated both cell-mediated and humoral immunity, as demonstrated by the higher expression of genes encoding CD4 and CD8 surface receptors in comparison with the control group. The expression of the gene encoding the CD4 receptor peaked at 7 dpi in all birds receiving herbal extracts, in particular in the group administered *Aloe vera* extract at 300 mg/kg BW. Similar results were reported by Vahedi et al. (2011) who observed increased proliferation of CD4^+^ and CD8^+^ lymphocytes in rabbits receiving *Aloe vera* extract [43]. Interestingly, the gene encoding receptor CD4 was least expressed in pigeons receiving the *Aloe vera* extract at 500 mg/kg BW (below control group levels at 4 and 14 dpi), which indicates that higher doses of the *Aloe vera* extract deliver immunosuppressive effects. The above phenomenon was also noted in analyses of CMI because the expression of the gene encoding the CD8 receptor was lower in the above group than in the remaining experimental groups and the control group. However, the observed differences were significant only relative to group D (licorice extract dose of 500 mg/kg BW) (Fig. 1). The expression of the gene encoding the CD3 receptor was similar to the expression of the gene encoding the CD8 receptor because the CD3 receptor is present on all T cells and, together with the T-cell receptor (TCR), it forms the TCR-CD3 complex responsible for antigen recognition and the transmission of the T-cell activation signal [14]. In view of the above, the increase in the expression of the gene encoding the CD4 receptor or the CD8 receptor should be correlated with a similar increase in the expression of the gene encoding the CD3 receptor. Such a correlation was observed in this study. The decrease in the expression of all analyzed genes at 14 dpi can be attributed to the weakening of immune responses after the elimination of immunomodulatory additives from pigeon diets.

Somewhat different results were noted in pigeons that received immunomodulatory feed additives and were then experimentally infected with PPMV-1. A clear increase in the expression of the gene encoding the CD4 receptor was observed, and it was correlated with an increase in the expression of the gene encoding the CD3 receptor in birds receiving both doses of the *Aloe vera* extract relative to control group birds and birds receiving licorice extracts. The highest correlation (*P* = 0.011) was noted between groups B1 (*Aloe vera* dose of 500 mg/kg BW) and C1 (licorice extract dose of 300 mg/kg BW) (Fig. 2). No such correlations were observed in an analysis of cytotoxic lymphocytes.

The immune response to a viral infection also leads to an increase in the synthesis of interferons which target viruses directly by inhibiting their protein synthesis or indirectly by activating defense mechanisms [41]. In the current study, special attention was paid to the expression of the gene encoding IFN-γ because this protein plays a significant role in both immediate and long-term immune responses to a viral infection. IFN-γ is produced mainly by CD4^+^ and CD8^+^ T cells. This cytokine is also secreted by B cells, NK cells, NKT cells and professional antigen-presenting cells [33]. In our study, the expression of the gene encoding IFN-γ increased in all birds from the inoculated groups, which can be directly linked with experimental inoculation. Higher expression of the above gene in the groups infected with PPMV-1 and administered herbal extracts than in the control group (K1) could also be attributed to the immunomodulatory properties of *Aloe vera* and licorice extracts. Similar results were reported by Utsunomiya et al. (1997) in whose study, glycyrrhizin stimulated T cells to produce IFN-γ in mice infected with the influenza A virus [42]. In our study, despite an absence of significant differences between groups, the gene encoding IFN-γ was most highly expressed in pigeons administered *Aloe vera* extract at 500 mg/kg BW, which is partially consistent with the expression of the genes encoding CD3 and CD8 receptors (Fig. 2). The highest expression of the gene encoding IFN-γ in group B1 should be associated with the immune response to the viral infection, which was exacerbated by the immunomodulatory effect of *Aloe vera* extract. The PPMV-1 strain used in the study was pathogenic, and pathogenic paramyxoviruses are most potent in stimulating interferon synthesis [20, 24].

The humoral immune response is the last stage of the immune response to an infection, where M class antibodies are produced first and constitute the largest group of immunoglobulins [19]. In the present study, the percentage of IgM^+^ B cells was higher in the groups administered both *Aloe vera* and licorice extracts than in the control groups at 7 dpi. However, the noted differences were not significant, probably due to high values of standard deviation (Fig. 3). Therefore, the influence of the tested herbal extracts on humoral immunity could not be confirmed despite the fact an increase in the percentage of anti-NDV antibodies after the administration of *Aloe vera* extract has been reported by other authors [26]. However, in a study of rabbits, the *Aloe vera* extract did not influence serum immunoglobulin levels [43], which is consistent with our findings.

## Conclusion

It can be concluded that both *Aloe vera* and licorice extracts exerted immunomodulatory effects, but their efficacy was clearly correlated with dose and the health status of the analyzed birds. In healthy pigeons, herbal extracts influenced both humoral and cell-mediated immune responses, but the immunostimulatory effects of *Aloe vera* were observed only in birds receiving a lower dose of the extract. A higher dose of *Aloe vera* extract exerted immunosuppressive effects on pigeons not infected with PPMV-1, and it exerted immunostimulatory effects on infected pigeons. The above correlations should be taken into consideration during the administration of *Aloe vera* extracts to birds.

## Abbreviation

BW: Body weight
cDNA: Complementary DNA
CFU: Colony-forming unit
CMI: Cell-mediated immunity
DPI: Days post-inoculation
GAPDH: Glyceraldehyde 3-phosphate dehydrogenase
IFN-ɣ: Interferon ɣ
IgM: Immunoglobulin M
IL: Interleukin
NDV: Newcastle disease virus
PBS: Phosphate-buffered saline
PCR: Polymerase chain reaction
PPMV-1: Pigeon paramyxovirus type 1
PRR: Pattern recognition receptors
SPF: Specific pathogen free
TLR: Toll-like receptors
